# Prophage ϕSA169 Enhances Vancomycin Persistence in Methicillin-Resistant *Staphylococcus aureus* (MRSA)

**DOI:** 10.3390/antibiotics14020191

**Published:** 2025-02-13

**Authors:** Yi Li, Andrew D. Berti, Wessam Abdelhady, Yan Q. Xiong

**Affiliations:** 1 The Lundquist Institute for Biomedical Innovation at Harbor-UCLA Medical Center, Torrance, CA 90502, USA; yi.li@lundquist.org (Y.L.); wabdel@lundquist.org (W.A.); 2 Department of Pharmacy Practice, Wayne State University College of Pharmacy and Health Sciences, Detroit, MI 48201, USA; andrew.berti@wayne.edu; 3 David Geffen School of Medicine at UCLA, Los Angeles, CA 90095, USA

**Keywords:** prophage, vancomycin persistence, MRSA endovascular infections

## Abstract

**Background:** Persistent methicillin-resistant *Staphylococcus aureus* (MRSA) endovascular infections present a significant clinical therapeutic challenge. Prophages are increasingly recognized as important genetic factors influencing the pathogenicity of *S. aureus*, yet their role in antibiotic persistence in MRSA remains underexplored. Our previous work demonstrated that prophage ϕSA169 promotes vancomycin (VAN) persistence in an experimental model of endocarditis caused by MRSA strains with a clonal complex (CC) 45 genetic background. However, it is unknown whether this persistence-promoting effect of ϕSA169 extends to other clinically relevant MRSA lineages. This study aims to elucidate the role of ϕSA169 in influencing VAN persistence across diverse MRSA genetic backgrounds. **Methods:** A pilot analysis of clinical data suggested that patients infected by MRSA containing ϕSA169-like prophage appear to have worse clinical outcomes. Thus, we lysogenized representative clinical resolving bacteremia (RB) MRSA strains with ϕSA169 and evaluated phenotypes closely associated with VAN persistence, including VAN susceptibility, biofilm formation, and the efficacy of VAN treatment in an experimental infective endocarditis (IE) model. Each ϕSA169 lysogenic strain was compared to its isogenic MRSA parental counterpart. **Results:** ϕSA169 lysogeny significantly promotes biofilm formation and enhances survival to VAN exposure under human-mimicking conditions for RB strains from CC5 and CC30. ϕSA169 lysogeny significantly reduces VAN effectiveness in the IE model due to RB lysogen from CC5 despite no detectable impact on VAN MICs. **Conclusions:** These results indicate that ϕSA169 promotes VAN persistence across clonal backgrounds, likely through biofilm formation and VAN tolerance. Targeting prophage could provide new strategies to combat persistent MRSA infections.

## 1. Introduction

Prophages represent a significant amount of the *Staphylococcus aureus* genome, accounting for up to 10% of its total genetic content [1,2,3]. It is estimated that approximately 90% of clinical *S. aureus* isolates harbor at least one prophage, with certain strains carrying multiple prophages [1,2,3]. Many prophages encode potent virulence factors that influence pathogenicity, antibiotic resistance, and immune evasion, thereby providing *S. aureus* with traits essential for adaptation and survival in stressful environments, such as during antibiotic exposure [4,5,6]. Such virulence factors include panton–valentine leucocidin (*pvl*), chemotaxis inhibitory protein (*chip*), staphylokinase (*sak*), and enterotoxins (*sea*, *selk2* and *selp*) [7,8,9]. Importantly, prophages serve as a primary mechanism for gene transfer between *S. aureus* strains, driving extensive genetic diversity and facilitating adaptation to diverse host conditions [10,11,12]. Thus, prophages are central to the success of *S. aureus* as a pathogen. Despite their critical role in pathogenicity, studies exploring interactions between prophages and the antibiotic persistence of MRSA during antibiotic therapy are limited.

In our previous work, we identified a novel prophage, ϕSA169, in a clinical persistent bacteremia (PB) MRSA strain (300-169) from CC45 [13]. PB is defined as ≥5 days of positive blood cultures despite appropriate antibiotic therapy [14]. This prophage was absent in a genetic-background-matched resolving bacteremia (RB) MRSA isolate (301-188) [13]. RB is defined as the initial positive blood cultures becoming negative within 2–4 days of antibiotic therapy [14]. Lysogenization of the RB MRSA strain 301-188 with ϕSA169 induced in vitro and in vivo phenotypic and genotypic characteristic changes in VAN persistence, closely resembling those observed in the PB MRSA strain 300-169 [15]. These findings suggest that ϕSA169 plays a significant role in promoting VAN-persistent outcomes in MRSA endovascular infections. However, this role was initially observed only in MRSA strains of the CC45 lineage, leaving questions about whether ϕSA169 may confer similar effects on VAN persistence in other *S. aureus* genetic backgrounds. We hypothesize that ϕSA169 contributes to VAN persistence in MRSA strains, irrespective of their genetic backgrounds. To test the hypothesis, we aim to determine whether ϕSA169 exerts the same effects on VAN persistence by lysogenizing a diverse collection of clinical RB MRSA strains with ϕSA169, thereby solidifying the role of ϕSA169 in antibiotic persistence in MRSA.

## 2. Results

### 2.1. Patients Infected by MRSA Containing ϕSA169-like Prophage Appear to Have Worse Clinical Outcomes

The impact of ϕSA169 on VAN persistence in MRSA has been demonstrated in our previous study using a pair of PB and RB strains from CC45. However, the broader impact of ϕSA169-like prophage on clinical cases remained unclear. To determine the clinical significance, if any, of infection with MRSA carrying the ϕSA169-like prophage, we conducted a pilot retrospective study using a dataset of pre-existing, de-identified information linking bacterial isolates with clinical outcomes. Our outcome of interest was a composite of PB and 30-day mortality. Of the 200 consecutive encounters queried, 122 were from unique patients with isolates containing paired whole genome sequence data and thus considered for further analysis. Of the 122 clinical isolates, 12 (10%) contained a prophage disrupting the *yfkAB* locus. These *yfkAB*-disrupting prophages were between 81% and 87% similar to ϕSA169 across the entire prophage length compared to uniformly negative alignment scores for prophages integrated at other loci. The two groups were well balanced at baseline by age (59 [52–72] vs. 56 [45–66], median [interquartile range], *p* = 0.46), gender (50% male vs. 63% male, *p* = 0.54), and by clinical isolate lineage (75% CC8 vs. 59% CC8, *p* = 0.19). Patients with isolates containing the *yfkAB*-disrupting prophage were more likely to experience PB or die compared to those from the cohort whose isolates had an intact *yfkAB* locus (92% vs. 55%, *p* = 0.01). Since VAN is typically the initial therapy of choice at our medical center, these preliminary clinical outcome findings suggest that ϕSA169-like prophages can contribute to PB.

### 2.2. Successful Construction of Lysogens

**Figure 1:**
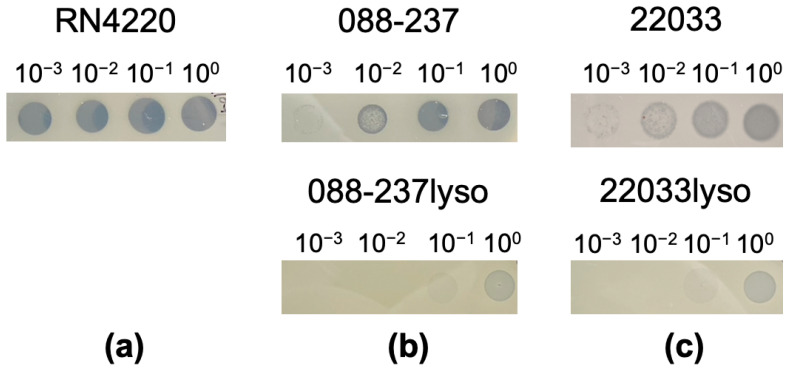
Superinfection immunity test of study strains: *S. aureus* RN4220 served as a negative control (**a**), MRSA RB 088-237 parental and 088-237lyso strains (**b**), and MRSA 22033 parental and 22033lyso strains (**c**) using serial diluted ϕSA169 lysates. Clear zones (plaques) indicate the strain is susceptible to ϕSA169 lysates, suggesting the absence of ϕSA169 in the strain.

**Figure 2:**
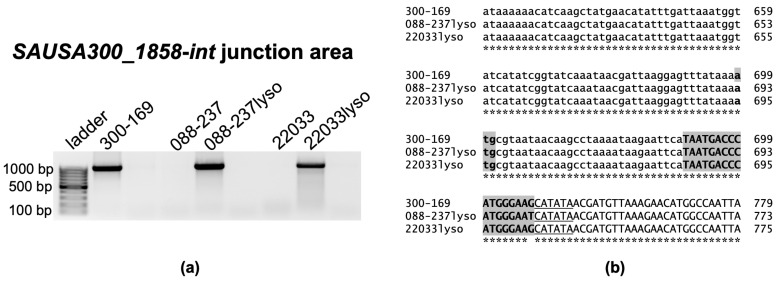
Gel electrophoresis of PCR products for the junction region of the prophage integration site at *SAUSA300_1858-int* (**a**) and the sequencing results of the PCR products for the junction region of the prophage integration site at *SAUSA300_1858-int*, aligned by Clustal Omega (**b**). Displayed are the 3′ end of the prophage integrase and the modified *yfkAB* 5′ sequence. Lowercase: sequence of prophage integrase gene (*int*), lowercase with gray background: new *yfkAB* start codon after prophage integration, uppercase: sequence of *yfkAB*, uppercase with gray background: *attB* core sequence, underlined: *attB*-flanking “CATAWA” element, *: indicates perfect sequence agreement in 3/3 isolates at the indicated position.

Three clinical RB MRSA isolates, 088-237, 30568, and 22033, were selected to study the impact of phage ϕSA169 on VAN persistence. MRSA RB strains 088-237, 30568, and 22033 were selected as a representative of MRSA CC5, CC8, and CC30 from our strain collection, respectively [16,17]. MRSA isolates from CC5, CC8, and CC30 are among the most prevalent causes of healthcare-associated invasive infections [18]. All three MRSA strains have an intact *yfkAB* gene (*SAUSA300_1858*) which contains the insertion site (*attB*) of ϕSA169 [15], indicating the capability of the strains to accommodate prophage in the genome. Despite this apparent phage susceptibility, repeated attempts to generate a ϕSA169 lysogen in RB MRSA 30568 were unsuccessful, and we decided to proceed with representatives of the other two lineages. To confirm lysogenization, we employed a superinfection immunity assay, a hallmark feature of lysogeny, which demonstrates resistance to infection by homologous phages [19]. As shown in Figure 1, serially diluted ϕSA169 lysates failed to form clear plaques on the bacterial lawns of the lysogenized strains (088-237lyso and 22033lyso). In contrast, plaques were observed on the prophage-free RN4220 strain and the two recipient RB MRSA parental strains (088-237 and 22033). Additionally, clear plaques were formed on the RN4220 lawn when exposed to the phage lysate at low concentrations but not on the lawns of RB MRSA parental strains. This discrepancy might be attributed to the dysfunction of Sau1 in RN4220 that allows for acceptance of foreign phage DNA [20]. These findings indicate that both lysogenized strains exhibited resistance to ϕSA169 infection, suggesting the integration of ϕSA169 into their genomes. To further confirm the integration of the ϕSA169 genome into the chromosome of the two RB MRSA recipient strains, we performed a PCR analysis targeting the *attL* prophage insertion site within *yfkAB* (*SAUSA300_1858*). The target region was successfully amplified from the lysogenized strains, as well as the ϕSA169-positive MRSA PB 300-169 strain, whereas no amplification was observed in the parental RB MRSA strains (Figure 2a). These results strongly support the integration of the ϕSA169 genome in the lysogenized strains. Additionally, sequencing of the *attL* PCR products spanning the *SAUSA300_1858* and gene-encoding integrase of the ϕSA169 (*int*) junction confirmed that the nucleotide sequences from the lysogenized strains were identical to the corresponding region in the CC45 PB MRSA 300-169 strain (Figure 2b). Together, these phenotypic and genotypic validations provide evidence that the ϕSA169 genome was successfully lysogenized into the 088-237 and 22033 RB MRSA strains.

### 2.3. Prophage ϕSA169 Significantly Enhances Biofilm Formation in MRSA Strains

**Figure 3:**
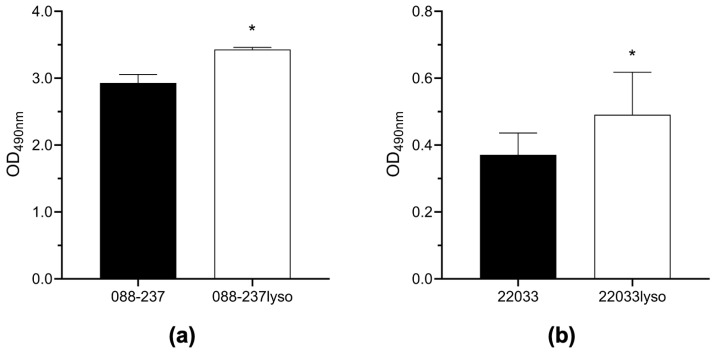
Biofilm formation of 088-237 (**a**) and 22033 (**b**) and their isogenic ϕSA169 lysogens. * *p* < 0.05 vs. their respective parental strains.

Given the critical role of biofilm formation in MRSA antibiotic persistence [21,22], enabling bacterial survival and resistance under hostile conditions, we assessed the impact ϕSA169 on biofilm-forming capacity of the two MRSA RB strains lysogenized with ϕSA169 in comparison to their respective parental strains. As shown in Figure 3, the ϕSA169 lysogens (088-237lyso and 22033lyso) exhibited a significant increase in biofilm formation compared to their respective parental counterparts. These findings indicate that the integration of ϕSA169 enhances biofilm formation, potentially contributing to the VAN persistence of these strains.

### 2.4. Prophage ϕSA169 Significantly Enhances MRSA Survival During VAN Exposure

**Figure 4:**
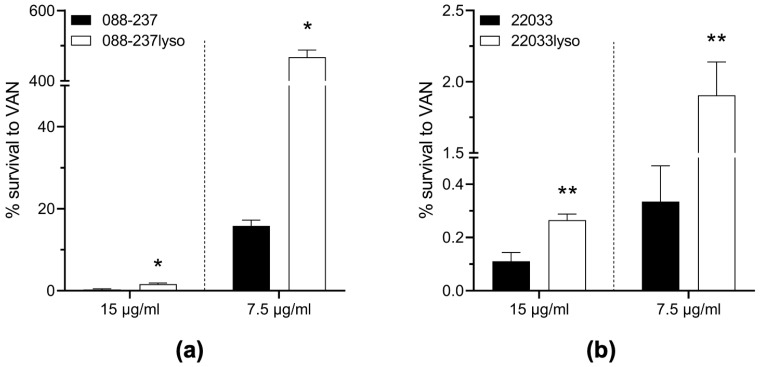
Viability changes after 24 h VAN exposure at 15 μg/mL (mimicking scenarios in human) and 7.5 μg/mL for 088-237 (**a**) and 22033 (**b**) strain sets. * *p* < 0.05, ** *p* < 0.01 vs. their respective parental strains.

To investigate the impact of ϕSA169 on VAN susceptibility, we first determined the VAN MICs on the study strains. Both ϕSA169 lysogens of 088-237 and 22033 exhibited VAN susceptibility with MICs of 1.0 μg/mL (Table 1). Importantly, these MICs were identical to those of their respective parental strains, indicating that ϕSA169 integration does not alter VAN MICs under standard in vitro testing conditions. However, persistent MRSA infections, particularly endovascular syndromes, are frequently associated with VAN treatment failures, even in infections caused by MRSA strains categorized as VAN-susceptible by CLSI breakpoints. To mimic the conditions of human endovascular infections, we assessed in vitro VAN-killing activity under high-inoculum (~10^8^ CFU/mL) mirroring MRSA density in cardiac vegetations in the IE and at two VAN concentrations: 15 μg/mL, a clinically relevant serum trough concentration obtained with standard VAN dosing in severe MRSA infections, as well as 7.5 μg/mL to assess VAN killing at subtherapeutic concentrations that nevertheless remain above the MIC of the study strains, including the ϕSA169-lysogenized strains of 088-237 and 22033 and their respective parental strains. The results revealed that both lysogens exhibited significantly enhanced survival during VAN exposure compared to their respective parental strains (Figure 4). This effect was particularly prominent with 088-237 strain set at 7.5 µg/mL VAN, where the wildtype parental strain demonstrated over 80% reduction in viability, whereas the lysogenized counterpart (088-237lyso) maintained substantial growth, underscoring reduced VAN-mediated killing in the presence of ϕSA169 (Figure 4a). These results suggest that ϕSA169 integration reduces VAN-killing activity under conditions mimicking endovascular infections. This reduced susceptibility likely facilitates incomplete bacterial clearance, contributing to VAN persistence in MRSA endovascular infections. Importantly, although lysogeny in both strain backgrounds did result in a statistically significant decrease in VAN killing, the magnitude of the effect was distinct between the two lineages, suggesting that CC30-specific factors may partially compensate for ϕSA169-mediated VAN persistence.

### 2.5. Prophage ϕSA169 Promotes MRSA VAN Persistence During Treatment in an Experimental IE Model

**Figure 5:**
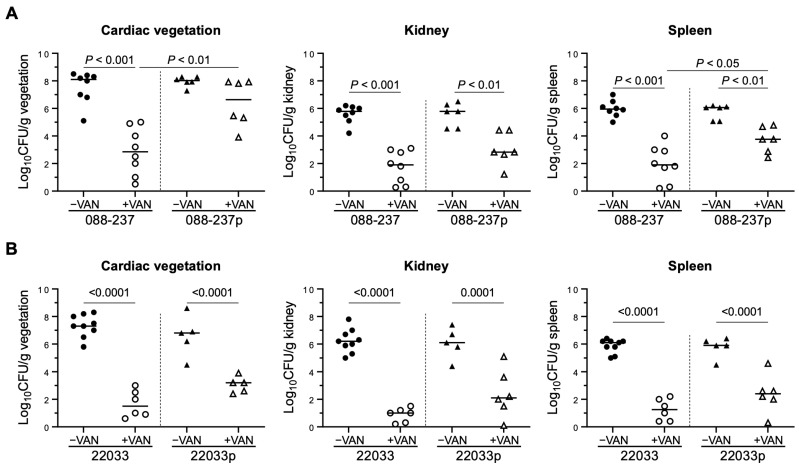
MRSA densities of 088-237 (**A**) and 22033 (**B**) strain sets in cardiac vegetation, kidney, and spleen in the IE model with and without VAN treatment. Each dot represents one animal. Horizontal black bars indicate the mean of MRSA densities.

To further investigate the impact of ϕSA169 on CC5 and CC30 RB MRSA strains in vivo, we utilized a rabbit IE model to assess virulence and response to VAN therapy of the lysogen strains. In the absence of VAN treatment, no statistically significant differences in intrinsic virulence were observed between the lysogens and their parental strains, as indicated by comparable MRSA counts in cardiac vegetations, kidneys, and spleens (Figure 5). However, in VAN-treated animals, a notable pattern in target tissue MRSA densities emerged, reflecting the in vitro VAN viability results. Specifically, rabbits infected with strain 088-237lyso and treated with VAN exhibited significantly higher bacterial densities in the cardiac vegetations and spleen compared to those infected with the parental strain 088-237 (Figure 5A). In contrast, while there was a trend toward increased bacterial density in the target tissues, no statistically significant differences were observed in any end organs between rabbits infected with strain 22033lyso and treated with VAN compared to those infected with the parental strain 22033 (Figure 5B). This is consistent with the more modest magnitude differences in viability at 7.5 µg/mL demonstrated in Figure 4 and reflective of lineage-specific differences in ϕSA169-mediated VAN persistence. While we cannot rule out the possibility of 22033lyso contributing to VAN persistence at lower concentrations, these systemic exposures would not be physiologically relevant. Overall, these findings indicated that the presence of ϕSA169 is associated with reduced VAN efficacy and enhanced VAN persistence in the IE model in a lineage-specific manner, suggesting a potential role for ϕSA169 in promoting persistence to VAN therapy in MRSA endovascular infections in either the CC5 or the CC45 lineage but not in the CC30 lineage.

## 3. Discussion

In this study, we investigated the impact of the novel prophage ϕSA169 on key phenotypic traits of MRSA strains with CC5 and CC30 genetic backgrounds, with a particular focus on its role in VAN persistence in MRSA endovascular infections. Our findings are consistent with previous research on CC45 MRSA [15] and support our hypothesis, demonstrating that ϕSA169 enhances MRSA biofilm formation, promotes surviving under VAN exposure, and contributes to persistence during VAN treatment in an experimental IE model. However, the extent of ϕSA169’s impact appears to be dependent on the specific MRSA strain/lineage. This study extends our understanding of ϕSA169 by revealing its effects across diverse MRSA strains with varying genetic backgrounds, highlighting the broader significance of this prophage in antibiotic persistence in MRSA endovascular infections.

Our prior in vitro and in vivo findings prompted us to look for ϕSA169-like prophages in whole genome sequence data from a pre-existing de-identified dataset of linked clinical outcomes. In this cohort of sequential patients, infection with an isolate that contains a ϕSA169-like prophage was associated with a significantly higher likelihood of PB or mortality, further reinforcing the contribution of ϕSA169 to poor patient outcomes. We do note that the relatively low prevalence of the ϕSA169-like prophage, the high baseline rate of PB, and the lack of baseline disease severity information in this cohort are significant limitations to any interpretation.

Based on the low prevalence of CC45 in the clinical dataset, we decided to explore the impact of ϕSA169 lysogeny in other staphylococcal lineages. Consistent with our previous findings [15], we observed that ϕSA169 does not alter the VAN MICs in lysogenized MRSA strains, indicating that this prophage does not confer direct VAN resistance under standard MIC assay conditions. However, our results revealed that ϕSA169 lysogens exhibit enhanced survival in VAN-killing assays under conditions mimicking human endovascular infections. The increased survival during VAN exposure is likely linked to the high MRSA initial inoculum, which may enhance biofilm formation and reduce antimicrobial efficacy, a well-established mechanism enabling VAN persistence [26]. Supporting this, we observed increased biofilm formation in both lysogenized strains, reinforcing the association between ϕSA169-driven biofilm production and VAN persistence described in our previous study [15]. Furthermore, this study extends the impact of ϕSA169 on biofilm formation to MRSA strains from genetic lineages CC5 and CC30, highlighting the broader influence of ϕSA169 across diverse MRSA genetic backgrounds.

Of particular interest, the in vivo results are also consistent with previous findings [15]. In the absence of VAN treatment, animals infected with lysogens or parental strains exhibited comparable MRSA burdens in cardiac vegetations, kidneys, and spleens, indicating that ϕSA169 does not significantly impact MRSA virulence under these conditions. However, with VAN treatment, the lysogenized RB strain from CC5 demonstrated markedly higher bacterial densities in both vegetation and spleen compared to their respective parental counterparts, despite identical VAN MICs between the lysogenized and their respective parental strains. The findings underscore the role of ϕSA169 in promoting MRSA VAN persistence during therapy, emphasizing its potential contribution to the challenge of treating persistent MRSA endovascular infections.

In addition to CC5 and CC30, CC8 is also a widely prevalent *S. aureus* lineage of significant clinical importance [27,28]. CC8 strains are responsible for the majority of community-associated MRSA infections and a substantial proportion of healthcare-associated MRSA infections [29,30,31]. We do note that the most prevalent lineage in the clinical outcome data was CC8, suggesting that ϕSA169 may also contribute to PB in CC8 MRSA. In our study, we attempted to construct ϕSA169 lysogens in the RB MRSA strain 30568, which belongs to CC8. However, these efforts were unsuccessful. As strain 30568 is a clinical isolate, it likely harbors other prophages that may confer resistance to ϕSA169 infection. In future studies, we plan to select alternative CC8 RB MRSA strains to further investigate the impact of ϕSA169. We also recognize that using a single RB MRSA isolate from each of two distinct genetic backgrounds (CC5 and CC30) provides only a preliminary insight into the potential effects of ϕSA169 on VAN persistence in MRSA. This inherently limits the scope of our findings, as the small sample size prevents us from drawing robust conclusions. To address this limitation, future studies will incorporate a larger number of MRSA isolates from CC5, CC30, and other prevalent lineages to validate the findings of the current study.

The molecular mechanisms underlying ϕSA169-associated VAN persistence in MRSA strains from different genetic backgrounds were not comprehensively explored in the recent study. While ϕSA169 demonstrated a contribution to VAN persistence in MRSA from CC5 and CC30, its impact was less pronounced compared to MRSA from CC45. This variation likely reflects genetic differences among MRSA lineages. Regardless of genetic background, the ϕSA169 integration process, including the interruption of the *yfkAB* (*SAUSA300_1858*) and the transcription of ϕSA169 genes, may contribute collectively to VAN persistence. In our recent work, we studied one of the ϕSA169 genes, *gp05*, and elucidated its role in promoting VAN persistence in MRSA. Future investigations will focus on identifying specific genetic factors within diverse MRSA lineages that are influenced by ϕSA169 and further characterizing the contributions of ϕSA169 genes. These efforts aim to provide a more comprehensive understanding of the mechanisms by which ϕSA169 influences VAN persistence in MRSA.

## 4. Materials and Methods

### 4.1. Paired Clinical Isolate-Clinical Outcomes Data

A minimal de-identified dataset was obtained from existing data, consistent with Wayne State University IRB 014518M1E and Detroit Medical Center IRB 14539 approval. The data included a unique identifier that could be linked to a biobanked clinical isolate but not to a patient’s record, age, gender, or microbiological data, including dates of blood cultures, date of discharge, repeat infectious disease encounter within 6 months (yes/no), and 30-day mortality. Whole genome sequence information for clinical isolates is available under PRJNA1048360. Clonal complex assignment was based on multi-locus sequence typing performed using pubmlst.org.

### 4.2. Bacterial Strains and Growth Medium

Bacterial strains used in this study are listed in Table 1. All the strains were routinely recovered from −80 °C stock by plating on 5% blood agar plates (Hardy Diagnostics, Santa Maria, CA, USA) and incubating aerobically at 37 °C. Single colonies were subsequently selected and cultured aerobically at 37 °C in tryptic soy broth (TSB; Becton, Dickinson and Company, Franklin Lakes, NJ, USA) or on tryptic soy agar (TSA) plates if not otherwise specified.

### 4.3. Isolation and Amplification of Phage ϕSA169

PB MRSA 300-169 was cultured to stationary phase and then supplemented with mitomycin C (1 μg/mL; Sigma-Aldrich, St. Louis, MO, USA), an agent used to induce prophage release [32]. The supernatant of the cell culture was filtered through a 0.22 μm filter (MilliporeSigma, Burlington, MA, USA) to remove bacterial cells [33]. The phage was then propagated and amplified in the prophage-free *S. aureus* strain RN4220 using a well-established double-layer technique and incubated at 37 °C until phage plaques developed [34]. In brief, the *S. aureus* cell culture was mixed with phage lysate to facilitate phage infection, then mixed with liquid top agar (TSB containing 0.5% agar and 1 mM calcium chloride), poured over a TSA plate, and incubated overnight at 37 °C once the top layer agar was solidified. The amplified phage was harvested by soaking the plates with phage plaques with phage buffer (60 mM β-glycerol phosphate disodium salt, 1 mM magnesium sulfate, 80 mM sodium chloride, 1% *w*/*v* gelatin, 4 mM calcium chloride) and filtered through 0.22 μm filter to remove bacterial cells.

### 4.4. Lysogen Construction and Superinfection Immunity Test

Lysogens of the study strains were constructed following the previously established assay [35,36]. In brief, cell cultures of the selected RB strains were plated on TSA plates seeded with phage (~10^9^ PFU/plate) incubated at 37 °C overnight. The surviving colonies on the plates, indicating that the bacteria evaded phage lysis, may harbor the prophage genome and potentially exhibit resistance to follow the phage-mediated lysis. Therefore, the surviving colonies were collected, cultured, and plated on TSA plates using the double-layer technique. Once the top layer agar solidified, lysates of ϕSA169 were serially diluted in the phage buffer and spotted onto the plates. The plates were then incubated at 37 °C, and the absence of clear phage plaques on the spotted areas indicated potential lysogens, as successful lysogens are resistant to superinfection by the same phage [37]. RN4220, a prophage-free strain that is susceptible to ϕSA169 infection, was included as a negative control.

### 4.5. Confirmation of Lysogens

To confirm successful lysogenization, colonies from the superinfection immunity assay were cultured (indicating the strain carries ϕSA169), and total genomic DNA was extracted using InstaGene^TM^ matrix (Bio-Rad, Hercules, CA, USA) according to the manufacturer’s instructions [38]. Primers were designed for polymerase chain reaction (PCR) to verify lysogen integration targeting the junction region of the prophage integration site at *SAUSA300_1858-int* (F: 5′-TACGACCCCACATTATCGGT-3′ and R: 5′-AACATTGGTTCGCACCTGT-3′) [15]. PCR products sizes were assessed by gel electrophoresis. The PCR products were sequenced by Azenta Life Sciences (Burlington, MA, USA), and the resulting sequences were aligned with the genome of PB 300-169 (Accession: JASL00000000) from GenBank nucleotide database to confirm the integration of the phage genome. The lysogens of the two RB strains were named 088-237lyso and 22033lyso (Table 1).

### 4.6. Determination of Antibiotics Minimum Inhibitory Concentrations (MICs)

VAN MICs were determined by a standard Etest method according to the manufacturer’s recommended protocols (BioMerieux, La Balme-les-Grottes, France).

### 4.7. Biofilm Formation

Biofilm formation of the study strains was performed under static conditions as previously described [39]. Briefly, MRSA cells from fresh culture plates were adjusted to a density of OD_600nm_ of 0.5 and diluted 1:100 into brain heart infusion (BHI) broth supplemented with 0.5% glucose. A total of 200 μL of the bacterial suspension was transferred into 96-well tissue culture plates and incubated statically for 18 h at 37 °C. Following incubation, the wells were gently washed with distilled water, air-dried, and stained with 0.1% safranin (*w*/*v* in distilled water). The adhering dye was dissolved in 30% acetic acid, and biofilm formation was quantified by measuring absorption at OD_490nm_.

### 4.8. In Vitro VAN Killing Activity

Overnight cultures of the study strains were washed, adjusted to OD_600nm_ of 1.0 in PBS, and diluted 1:10 into cation-adjusted Mueller–Hinton broth (MHB; Thermo Fisher Scientific, Waltham, MA, USA) to achieve an initial inoculum of ~10^8^ CFU/mL (similar MRSA density in cardiac vegetations in the IE) and exposed to VAN at 15 μg/mL (serum trough levels in human with standard VAN treatment in severe MRSA infections) or 7.5 μg/mL at 37 °C with shaking at 200 rpm for 24 h. Survival rates were assessed by the ratio of the MRSA-surviving cell population vs. the initial inoculum which were enumerated by dilution plating.

### 4.9. Experimental IE Model in Rabbits

To evaluate the impact of prophage ϕSA169 on in vivo virulence and VAN responsiveness, a well-established rabbit model of catheter-induced aortic valve IE was employed [26]. At 72 h post-aortic-catheterization, rabbits were infected intravenously (i.v.) with the study strains (~10^5^ CFU/animal, corresponding to the previously established 95% infective dose [ID_95_]) [26]. At 24 h post-infection, the animals were randomly assigned to either a control group (no therapy) or a treatment group receiving VAN (15 mg/kg, i.v. twice daily for 3 days), which represents a standard effective VAN dose in this experimental IE model with VAN-susceptible strains [26]. The control animals were sacrificed at 24 h post-infection to determine the MRSA tissue burden at the initiation of VAN treatment. The VAN-treated animals were euthanized at 24 h after the final dose to prevent VAN carryover effects. At sacrifice, cardiac vegetations, kidneys, and spleens were removed and quantitatively cultured to determine bacterial density [40]. MRSA counts in each target tissues were calculated as the mean log_10_ CFU per gram of tissue (±standard deviation [SD]) [26]. All rabbits were handled in accordance with the guideline set by the American Association for Accreditation of Laboratory Animal Care. The Institutional Animal Care and Use Committee (IACUC) of the Lundquist Institute at Harbor-UCLA Medical Center approved the animal studies.

### 4.10. Statistical Analysis

All in vitro experiments were performed in triplicates and repeated at least twice. A two-tailed Student *t*-test was employed to analyze the in vitro data and the in vivo MRSA counts in the target tissues, using Prism 9 (GraphPad Software, San Diego, CA, USA). A Mann–Whitney U test was applied to ordinal and continuous patient data, while a Chi-squared test was applied to categorical data, using Microsoft Excel (Microsoft Corporation, Redmond, WA, USA) with the RealStats plugin. *p* values < 0.05 were considered statistically significant.

## 5. Conclusions

In summary, this study highlights the pivotal role of ϕSA169 in facilitating persistent MRSA endovascular infections by enhancing biofilm formation and increasing survival during VAN exposure under conditions that closely mimic in vivo environments. Notably, these effects were observed across MRSA strains representing diverse CC types, despite identical VAN MICs between parental and its isogenic ϕSA169 lysogenized strains. These findings highlight ϕSA169 as a critical determinant influencing VAN responsiveness in the experimental endocarditis model. Future investigations will focus on elucidating the molecular mechanisms by which ϕSA169 modulates MRSA virulence factors and interacts with host immune responses to drive persistent MRSA endovascular infections.

## Figures and Tables

**Table 1:** *S. aureus* strains used in this study.

Strains	Characteristics	VAN MIC (μg/mL)	References
088-237	RB-MRSA, *agr-II*, SCC*mec* II, CC5	1.0	[16,23]
088-237lyso	088-237 lysogenized with prophage ϕSA169	1.0	This study
22033	RB-MRSA, *agr-III*, SCC*mec* IV, CC30	1.0	[17]
22033lyso	22033 lysogenized with prophage ϕSA169	1.0	This study
300-169	PB-MRSA, *agr-I*, SCC*mec* IV, CC45; donor of ϕSA169		[16,23]
RN4220	MSSA, NCTC8325-4, α-hemolysin negative, β-hemolysin positive		[24,25]

## Data Availability

Data are contained within the article.
